# Questions Left Unanswered: How the Brain Responds to Missing Information

**DOI:** 10.1371/journal.pone.0073594

**Published:** 2013-10-02

**Authors:** John C. J. Hoeks, Laurie A. Stowe, Petra Hendriks, Harm Brouwer

**Affiliations:** 1 Center for Language and Cognition Groningen, University of Groningen, Groningen, The Netherlands; 2 BCN Neuroimaging Center, University of Groningen, Groningen, The Netherlands; Baycrest Hospital, Canada

## Abstract

It sometimes happens that when someone asks a question, the addressee does not give an adequate answer, for instance by leaving out part of the required information. The person who posed the question may wonder why the information was omitted, and engage in extensive processing to find out what the partial answer actually means. The present study looks at the neural correlates of the pragmatic processes invoked by partial answers to questions. Two experiments are presented in which participants read mini-dialogues while their Event-Related brain Potentials (ERPs) are being measured. In both experiments, violating the dependency between questions and answers was found to lead to an increase in the amplitude of the P600 component. We interpret these P600-effects as reflecting the increased effort in creating a coherent representation of what is communicated. This effortful processing might include the computation of what the dialogue participant meant to communicate by withholding information. Our study is one of few investigating language processing in conversation, be it that our participants were ‘eavesdroppers’ instead of real interactants. Our results contribute to the as of yet small range of pragmatic phenomena that modulate the processes underlying the P600 component, and suggest that people immediately attempt to regain cohesion if a question-answer dependency is violated in an ongoing conversation.

## Introduction

During conversation, speakers and listeners act upon certain basic assumptions which enable them to communicate swiftly, and seemingly effortlessly [1]–[5]. If, for instance, someone asks a question, both speaker and hearer have knowledge of what would constitute a valid answer. To be more specific, a question can be said to impose constraints and create expectations regarding both the *information structure* (i.e., specifying what is given and what is new, and thus how the information contained in an utterance should be linked to the existing discourse representation) and the *content* of the answer. Consider for instance someone inquiring about the activities of two protagonists, ‘John’ and ‘Peter’:

1. What did John and Peter do?

On the level of information structure, this question introduces two entities that make them likely *topics* in the answer, where a topic can be loosely described as the entity about which the sentence imparts information [6]. On the content level, in turn, the question requires the answer to impart on the activities of these specific people (‘John’ and ‘Peter’), and not, for instance, about their respective spouses. Answer (2) satisfies both of these constraints.

2. John cleaned the house and Peter fixed the window.

In contrast, by leaving out information about the second protagonist, answer (3) violates expectations regarding both information structure and content. Utterance (3) is thus pragmatically infelicitous as an answer to question (1).

3. John cleaned the house.

If there is no additional information, and the answer consists of only this sentence, the person who posed the question is faced with the task of determining what the speaker meant to communicate by being incomplete. The speaker might, for instance, be taken to convey that Peter did nothing, that what he did was of no importance, or just that Peter is terribly lazy [1], [7]. The computation of such beliefs, and thus of a coherent mental representation of intended meaning, may require extensive pragmatic processing [Regel, Gunter, & Friederici [8] provide a similar argument on the computation of ironic meaning]. How the human language processor deals with this kind of processing is still poorly understood, and neurocognitive investigations of such phenomena are scarce.

This study presents two Event-Related brain Potential (ERP) experiments that examine the neural correlates of the pragmatic processes invoked by partial answers to questions. ERPs provide a means of disentangling different processes involved in online language comprehension, on the basis of the qualitatively different signatures they leave behind. There are many ERP studies on word- and sentence-level processing [Kutas, van Petten, & Kluender [9] provide an overview], but researchers have only recently started to use ERPs to investigate pragmatic processing [8], [10]–[13]. These latter studies provide evidence that pragmatic processes such as the computation of bridging inferences or of ironic meaning modulate the amplitude of the P600 component, a positive deflection of the ERP signal that usually peaks around 600 ms post stimulus onset.

Brouwer, Fitz, & Hoeks [14] have recently argued, on the basis of a thorough review of the ERP literature, that the P600 component is best defined as a family of late positivities that reflect the processing involved in the word-by-word construction, reorganization, or updating of a *mental representation of what is being communicated* (MRC)–see also [15], [16]. Different varieties of the P600-effect (in terms of electrophysiological properties like onset, amplitude, duration, and scalp distribution) are assumed to reflect different sub-processes of MRC construction. These sub-processes may include, among other things, the accommodation of new discourse referents, the establishment of relations between entities, thematic role assignment and revision, and for instance, the resolution of conflicts between different information sources (e.g., with respect to world knowledge). For instance, in the computation of bridging inferences, as in a sentence pair like “We went for a picnic. The beer was warm” [17], some of the sub-processes involved will concern the accommodation of the new discourse referent “The beer”. The computation of ironic meaning, on the other hand, may involve more sub-processes aimed at overcoming the conflict between the unfolding discourse and the ‘literal meaning’ of the ironic utterance—cf. “These artists are gifted!” in the context of a bad musical performance, see [8].

The present study investigates whether the processes invoked by partial answers to questions also produce an increase in P600 amplitude, which would provide strong support for the MRC hypothesis discussed above (i.e., P600 amplitude reflects ease of ‘making sense’).

## Results

### Experiment 1

In the first experiment, participants read short question-answer pairs that appeared word-by-word in the middle of a computer screen, and were occasionally asked to answer a comprehension question (see Procedure section below). During reading, brain activity of the participants was monitored through ERP recording. The question-answer pairs differed in the pragmatic felicity of the answer given the preceding question. We used two types of questions: ‘neutral’ questions like (4), which do not impose any strong constraints on the information structure of the answer, and questions such as (5) that require the answer to contain two topics in a so-called ‘contrastive topic’ information structure—cf. [18]. For the answers we used Dutch sentences containing NP-coordinations with a one-topic information structure, based on materials taken from [19]. In these sentences, the NP following the coordinator is temporarily ambiguous between being the subject of a new clause, or the object of the present clause. In Dutch and also in other languages, the object reading is preferred [20]. If such a one-topic answer follows a contrastive-topic question, as in (5), this constitutes a pragmatic violation: The question requires the answer to impart on the activities of two topics (“the mayor” and “the alderman”); in the answer these entities are mentioned, but only one of them (“the mayor”) turns out to be a topic.

It is important to note that in Dutch (unlike in English), the presence of the adverb at the end of the sentence unambiguously indicates that the ambiguous NP (“the alderman”) cannot be a topic, and that the sentence only has one topic. Thus at the adverb, the reader is confronted with a clear pragmatic violation. It should be noted, however, that whereas in the experiment there is no sentence following the partial answer, the missing information could in principle be given in a next sentence (e.g., question: “What did the mayor and the alderman do?”—answer: “The mayor praised the councilor and the alderman exuberantly. The alderman therefore thanked the mayor”). It would be interesting for a future experiment to manipulate the presence or absence of such an additional sentence.

4. *Neutral*

Q: Wat gebeurde er?

‘What happened?’

A: De burgemeester prees het raadslid en de wethouder uitbundig.

‘The mayor praised the councilor and the alderman exuberantly.’

5. *Violation*

Q: Wat deden de burgemeester en de wethouder?

‘What did the mayor and the alderman do?’

A: De burgemeester prees het raadslid en de wethouder uitbundig.

‘The mayor praised the councilor and the alderman exuberantly.’

#### Data analysis

**Figure 1:**
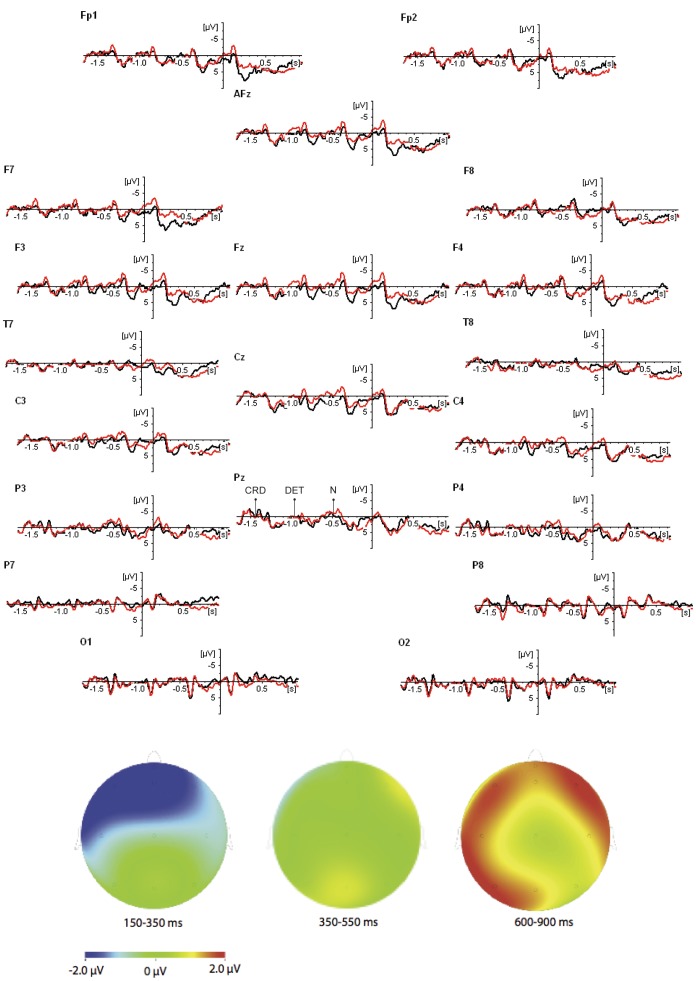
ERP waveforms for the two conditions in Experiment 1: Neutral (black line) and Violation (red line); topographic maps represent Violation minus Neutral; there is an extended pre-stimulus time-window in which the onset of the coordinator (CRD), determiner (DET), and noun (N) is indicated by arrows.

Participants were reading attentively, answering on average 85% (SD = 5.6) of the 35 content questions correctly. ERP waveforms were time-locked to the presentation of the critical adverb (“exuberantly”), see Figure 1.

Three time-windows for statistical analysis were chosen a priori: a window in which early effects might be observed (150–350 ms post-onset), a time-window in which possible N400 effects might be observed (350–550 ms post-onset), and a later time-window for a possible P600 (600–900 ms post-onset). For each of those intervals, average ERPs were computed for participant, condition and electrode separately. Prior to averaging, trials with ocular or amplifier-related artifacts were excluded from the analysis. For analysis purposes, three sets of electrodes were created: the three *prefrontal* electrodes FP1, FZA, and FP2; the two *occipital* electrodes O1 and O2; and the *main set* of the 15 remaining electrodes. For each of those sets, Repeated Measures ANOVAs were conducted with Violation (violation vs. neutral), Laterality and Anteriority as within-participant factors. In the prefrontal analysis, Laterality had 3 levels (i.e., left, midline, and right side of the scalp); in the occipital analysis, Laterality had 2 levels (i.e., left and right); for the main analysis, Laterality had 5 levels (far left, left, middle, right, far right), and Anteriority had 3 levels (anterior, central, and posterior). Where appropriate, the Huynh-Feldt correction was applied; corrected p-values will be reported with the original degrees of freedom. Only effects involving the factor Violation will be discussed.

*Non-standard baseline*. The pre-critical word (the ambiguous NP “the alderman”) in the target sentence is introduced in the context question of the violation condition, but not in the neutral condition. This gives rise to 1) a ‘repetition’ N400-effect, where the N400 in the violation condition is attenuated (as compared to the neutral condition) through word repetition; 2) a P600 effect, due to the fact that in the neutral condition “the alderman” is a new discourse entity, whereas in the violation condition it is already given [10], [14], [16]. As we wanted to avoid including these effects in our baseline, we chose a baseline on the coordinator “en” (“and”) that precedes the ambiguous NP (i.e., “… and the alderman exuberantly.”). Importantly, the presence of the positivity for the neutral condition may still affect the size of subsequent effects (if we assume that ERP waves are additive), as the violation condition starts out more negative than the neutral condition at some of the electrodes. Hence, our ‘early-baseline’ procedure may *overestimate* the size of negativities following the target word in the violation condition. Conversely, the fact that the violation condition is more negative to begin with may have decreased the amplitude of subsequent *positivities* associated with the violation condition. Thus, the early-baseline procedure may *underestimate* the size of any positivity following the target word in the violation condition.

#### Early Time Window (150–350 ms post-onset)

In the analysis of the main set of electrodes, there was a marginally significant interaction of Violation×Anteriority (F(2,30) = 3.1; p = .08). Follow-up analyses showed that this trend towards an interaction was most probably caused by a *negativity* for the violation condition (as compared to the neutral condition) that was largest at the frontal electrodes (violation: 2.1 µ*V* (SE = 0.6); neutral: 4.3 µ*V* (SE = 1.3)), smaller at central sites (violation: 2.7 µ*V* (SE = 0.7); neutral: 3.7 µ*V* (SE = 1.1)) and smallest at posterior electrodes (violation: 1.5 µ*V* (SE = 0.9)); neutral: 1.6 µ*V* (SE = 1.0)). At the *prefrontal* electrodes there was a marginally significant main effect of condition, again with violation being more negative than neutral (violation: 2.9 µ*V* (SE = 0.7); neutral: 5.3 µ*V* (SE = 1.1); F(1,15) = 3.9; p = .066). No effects were found in the analysis of the occipital electrodes.

#### N400 Time-Window (350–550 ms post-onset)

We did not find significant effects for the *main set* or for the *prefrontal* electrodes (all p-values>.27). At the *occipital* electrodes there was a marginally significant interaction of Violation×Laterality (F(1,15) = 3.5; p = .08), most probably because the positivity elicited in the violation condition was bigger at the left than at the right of the scalp (*Left*: violation: −0.28 µ*V* (SE = 0.9); neutral: −1.25 µ*V* (SE = 0.7); *Right*: violation: −0.65 µ*V* (SE = 0.8); neutral: −0.88 µ*V* (SE = 0.7)).

#### P600 Time-Window (600–900 ms post-onset)

The analysis on the main set of electrodes produced a significant interaction of Violation×nteriority×Laterality (F(8,120) = 2.5; p<.05). Follow-up analyses per level of Laterality suggested that this interaction was due to a specific pattern of results for electrodes situated at the far left (Violation×Anteriority: F(2,30) = 3.3; p = .059), indicating a *positivity* for the violation condition that was present at T7 (violation: 3.2 µ*V* (SE = 0.8); neutral: 1.6 µ*V* (SE = 0.7); F(1,15) = 4.5; p = .05) and P7 (violation: 1.1 µ*V* (SE = 1.1); neutral: −1.1 µ*V* (SE = 1.0); F(1,15) = 6.0; p<.05), but not at F7 (violation: 1.9 µ*V* (SE = 0.8); neutral: 1.9 µ*V* (SE = 1.3); F<1). At the other levels of Laterality, the violation condition was always more positive than the neutral condition, but none of these differences were significant (e.g., *left*: violation: 3.5 µ*V* (SE = 0.7); neutral: 1.7 µ*V* (SE = 1.0); *middle*: violation: 4.2 µ*V* (SE = .7); neutral: 3.1 µ*V* (SE = 1.3); *right*: violation: 4.3 µ*V* (SE = 0.7); neutral: 2.9 µ*V* (SE = 1.1); *far right*: violation: 3.1 µ*V* (SE = 0.5); neutral: 1.8 µ*V* (SE = 1.0); all p-values>.10). Analysis of the occipital electrodes showed a significant interaction of Violation×Laterality (F(1,15) = 2.8; p<.01), due to a larger positivity for the violation condition at the left side (O1: violation: 0.9 µ*V* (SE = 1.2); neutral: −0.7 µ*V* (SE = 1.2)) than at the right side (O2: violation: 0.6 µ*V* (SE = 1.1); neutral: 0.3 µ*V* (SE = 1.1)). At prefrontal electrodes, the violation condition (4.6 µ*V* (SE = 0.9)) was numerically more positive than the neutral condition (3.2 µ*V* (SE = 1.3)) but this difference did not reach significance (p>.12).

#### Discussion

Leaving a question partially unanswered gave rise to a significant, left-lateralized positive shift (600–900 ms after the onset of the target) which we interpret as a P600. The marginally significant effect at occipital electrodes in the “N400 time-window” suggests that this positivity already started earlier (350–550 ms post-onset), though with a different scalp distribution. These findings are consistent with the MRC hypothesis [14], where difficulties in creating a mental representation of language input are assumed to be reflected in (late) positivities. In addition to these positive effects, we found evidence for an early negativity (150–350 ms post-onset) with a frontal focus.

To start with this early negativity, Lau, Stroud, Plesch, and Phillips [21] reported a very similar finding in sentences containing a word category violation. They interpreted this effect as an Early Left Anterior Negativity or ELAN [22], [23]—see [24] for a critical review. ELAN effects are typically observed when the syntactic category of the presented word does not match reader expectation. In the present study, the question in the violation condition sets up the expectation that the two protagonists in the answer act as AGENTS, each involved in a separate event (e.g., an event depicting what “the mayor” did, and another event depicting what “the alderman” did). However, instead of with the expected verb, readers were presented with an adverb. This mismatch in category may have produced the ELAN-effect.

After reading the disambiguating adverb, the reader must deal with the fact that the mental representation of the sentence, based on the assigned information structure and on the assigned thematic roles, is partially incorrect and in need of revision: “the alderman” is (i) not a topic, but should become part of the comment, and (ii) not an AGENT but a PATIENT. However, this ‘local’ revision of the mental representation created thus far will not solve the larger, more ‘global’ problem of the missing information, which may require extensive pragmatic processing. That is, after revising the interpretation to reflect that “the alderman” is a PATIENT and part of a comment, rather than an AGENT and a topic, one is still faced with the problem of what is meant by leaving out information on what “the alderman” did. Hence, to regain a coherent interpretation of the unfolding dialogue, people have to update their mental representation to reflect, for instance, that the speaker has left out the information on purpose, for instance, to communicate that “the alderman” was passive, and did nothing at all.

In the present experiment, it is not possible to separate processes of local revision and global pragmatic processes, although one might be tempted to speculate that the local revision is reflected by the early positivity in the N400 window (the size of this effect was rather small, but possibly underestimated through the early baseline procedure, see Data Analysis section above), and the global, more pragmatic processing by the later positivity. In order to disentangle these processes, we conducted a second experiment, using target sentences which did not contain the ambiguous NP (“the alderman”), thereby eliminating the need for local revision.

### Experiment 2

In Experiment 1, the target sentence contained all discourse entities from the context question, only the information structure was manipulated. In Experiment 2, we entirely removed the ambiguous part of the target sentence, as shown in examples (6) and (7) below (the critical word is underlined).

6. *Neutral*

Q: Wat gebeurde er?

‘What happened?’

A: De burgemeester prees het raadslid.

‘The mayor praised the councilor.’

7. *Violation*

Q: Wat deden de burgemeester en de wethouder?

‘What did the mayor and the alderman do?’

A: De burgemeester prees het raadslid.

‘The mayor praised the councilor.’

Experiment 2 will thus provide an uncluttered view on how the brain deals with missing information. Comparing the results from this experiment with the results from Experiment 1 will enable us to estimate to what extent the local revision processes contribute to the positivity. And as the present manipulation does not involve any kind of category violation, we expect the early negativity found in Experiment 1 to disappear, which will give further credibility to the notion that the negativity observed was in fact an ELAN.

#### Data analysis

**Figure 2:**
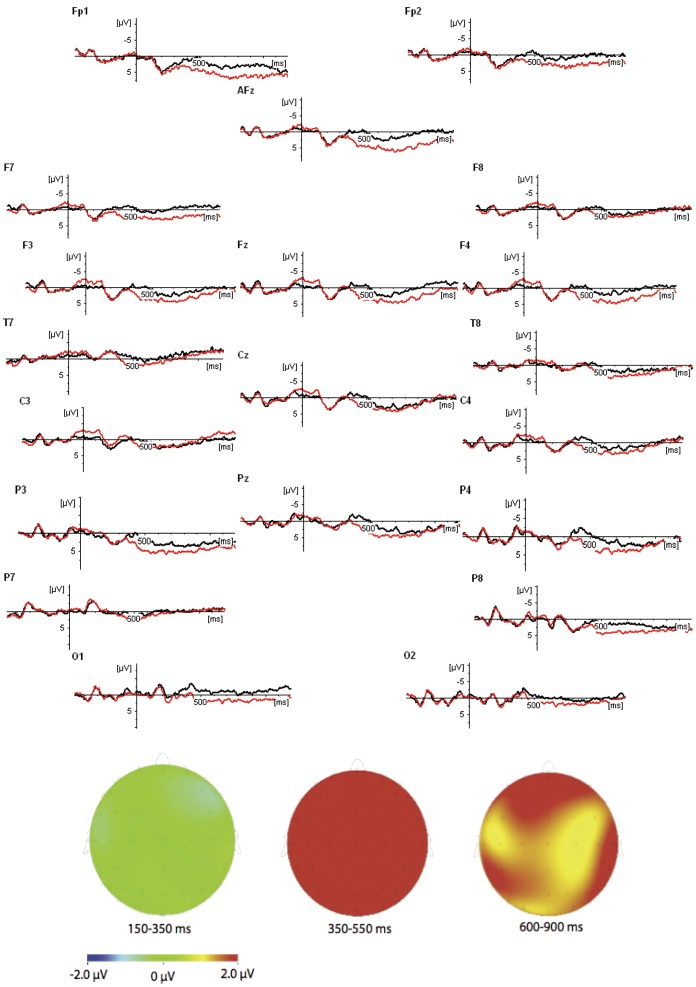
ERP waveforms for the two conditions in Experiment 2: Neutral (black line) and Violation (red line); topographic maps represent Violation minus Neutral.

Analysis methods were the same as for Experiment 1, except that we now used a more “standard” pre-stimulus baseline on the article (“the”) preceding the final noun (“councilor”). See Figure 2 for a graphical display of the resulting waveforms.

As in Experiment 1, we found a positivity (neutral condition more positive than violation condition) starting around the onset of the critical word. The cause of this effect is not completely clear, but it may reflect the ease with which new information is integrated into an existing mental representation. It is likely that this integration is easier when there is already some kind of representation, as in the violation condition, then when the mental representation has to be created from the start [10]. As in the previous experiment, this effect may *overestimate* any negativities and *underestimate* any positivities that follow the presentation of the target word in the violation condition. As we will see, this does not affect the interpretation of the results: there are no negativities (that may have been there spuriously), and the positive effects are rather large (and hence not eliminated by the baseline positivity).

#### Early Time Window (150–350 ms post-onset)

No significant effects were found in either of the analyses (all p-values>.14).

#### N400 Time-Window (350–550 ms post-onset)

We found a significant effect of Violation for the *main set* of electrodes (F(1,16) = 13.6; p<.005), with the violation condition being more *positive* (2.2 µ*V* (SE = 0.7)) than the neutral condition (−.05 µ*V* (SE = 0.4)). Additional significant main effects of Violation were found for the *prefrontal* electrodes (violation: 3.2 µ*V* (SE = 1.2); neutral: 0.3 µ*V* (SE = 1.0); F(1,16) = 9.0; p<.01) and the *occipital* electrodes (violation: 0.4 µ*V* (SE = 0.8); neutral: −1.7 µ*V* (SE = 0.6); F(1,16) = 6.0; p<.05).

#### P600 Time-Window (600–900 ms post-onset)

The main effect of Violation was significant for the *prefrontal*, electrodes (F(1,16) = 7.3; p<.05), again with the violation condition (2.2 µ*V* (SE = 0.7)) being more *positive* than the neutral condition (−0.05 µ*V* (SE = 0.4)). Main effects of Violation almost reached significance at the *main set* of electrodes (violation: 3.0 µ*V* (SE = 0.9); neutral: 1.5 µ*V* (SE = 0.5); F(1,16) = 4.1; p = .060) and at the *occipital* electrodes (violation: 1.8 µ*V* (SE = 0.6); neutral: −0.2 µ*V* (SE = 1.0); F(1,16) = 3.6; p = .076).

## Discussion

The results of both experiments show that the processes invoked by a partial answer to a question modulate the amplitude of the P600 component. In Experiment 1, all elements from the question were present in the answer, but with the wrong information structure. Disambiguation by the critical adverb required revision of the mental representation built so far, and instigated a change in information structure and thematic role assignment, as well as pragmatic processing regarding the ‘global’ issue of missing information. Therefore, the P600-effect that was found in Experiment 1 was argued to reflect both local revision and global pragmatic processing. In Experiment 2, by contrast, the entire ambiguous phrase was eliminated, making the resulting P600-effect a pure reflection of extensive (pragmatic) processing needed to make sense of the dialogue—see also [8] for a similar P600-effect for ironic versus non-ironic meaning.

The conclusion that both experiments produced a P600-effect fits very well with a recent neurocognitive framework proposed by Brouwer, Fitz, & Hoeks [14]—see also [15], [16]. Traditionally, the P600 component has been linked to syntactic processing [9], [25]. However, in the last decade, an increasing number of studies have found non-syntactic P600-effects [see [14], [26], [27] for overviews], and a few of these studies have even found P600-effects for specifically pragmatic phenomena such as bridging inferences [10]–[12] and the processing of irony [8]. On the basis of a thorough review of this literature, Brouwer and colleagues hypothesized that the P600 component is best defined as a family of late positivitities reflecting the different sub-processes involved in the word-by-word construction, reorganization, or updating of a mental representation of what is being communicated. Examples of these sub-processes are accommodating new discourse entities, establishing a relation between the entities and assigning them a thematic role, adding information to entities, revising already established relations, revising already assigned thematic roles, and resolving conflicts between information sources (e.g., with respect to world knowledge). In the case of incomplete answers, some of these processes might take the form of computing the meaning that is *implied* by the answer. For instance, if the question “What did the mayor and the alderman do?” is answered with “The mayor praised the councilor”, the observed P600-effect may reflect processes involved in constructing for instance the belief that the “alderman” has been idle, which is then added to the developing mental representation of the unfolding dialogue. To find out whether language users do indeed create such meanings, attempts should be made to investigate the mental representations that participants actually construct, for instance by having them report verbally on what they think is the intended meaning of an utterance, or, more covertly, to probe this representation in a priming paradigm.

An important difference in the results of the two experiments is that in Experiment 2, the P600-effect seems much more prominent from an early moment on (i.e., in the 350–550 ms time window) than in Experiment 1, though Experiment 1 does show a marginally significant positivity at the occipital electrodes. This difference in prominence of the P600 effect is not quite what would be expected. In Experiment 1, a local revision is required before more global (pragmatic) processing can commence. In Experiment 2, by contrast, there is no need for local revision. Hence, we would have expected Experiment to engender *more extensive* processing than Experiment 2. Close inspection of the data from Experiment 1, however, suggests that there is an early positivity that is likely to be underestimated as a result of the pre-existing effect at baseline. As we discussed above, in Experiment 1 we chose a non-standard baseline because of an attenuated N400 as well as a P600-effect on the pre-critical word in the neutral condition (see Data Analysis section of Experiment 1). The presence of a positivity on the pre-critical word in the neutral condition might have as a consequence that an actual P600-effect on the critical word in the violation condition is underestimated. Analysis of the three time-windows using a 100 ms *within*-stimulus baseline confirms that this might indeed be the case: in addition to the negativity in the 150–350 ms time window, and the positivity in the 600–900 ms time window, this analysis also reveals a significant positivity in the 350–550 ms time window (with a broad scalp distribution). If we accept this tentative evidence, it suggests that both experiments show a positivity starting as early as 350 ms after the onset of the critical word, signifying the effortful updating of a mental representation of what is said.

Another difference between the experiments is the difference in scalp distribution in the late 600–900 ms time window. In both experiments, the P600-effect showed a standard broad centro-parietal scalp distribution in the early 350–550 ms time window, but in the later 600–900 ms time-window, the P600-effect observed in Experiment 1 was strongly left-lateralized, whereas the P600-effect observed in Experiment 2 was more pronounced at prefrontal electrodes. Brouwer, Fitz, & Hoeks [14] argue that the sub-processes invoked in the creation of a coherent mental representation may be different in different phases and under different circumstances, and that these differences may be reflected in the P600 in terms of variations in ERP parameters such as onset, amplitude, duration, and scalp distribution. This suggests that the experiments may have invoked partly similar and partly different processes.

Exactly what processes are involved still needs to be determined. Van Petten & Luka [28] speculate that more frontally pronounced positivities reflect prediction errors, whereas more parietally pronounced positivities reflect reprocessing costs. It is difficult to see how this fits with our data, as one could argue that in both experiments there is some kind of prediction error (of the expected information structure), and there is a requirement for reprocessing (or at least more extensive processing in order to recover the intended meaning). Hence, it is unclear whether our data should evoke a frontal P600-effect, a parietal one, or some kind of combined positivity, reflecting both prediction error and reprocessing cost. Brouwer and Hoeks [15] have recently suggested an alternative explanation for the origin of characteristically different P600-effects. They hypothesize that the different sub-processes involved in MRC construction, elicit (late) positivities because these processes are implemented by neural generators residing in different parts of the left Inferior Frontal Gyrus (lIFG) (a conglomerate of areas around and including Broca's Area). This is consistent with the data of the present study. The experiments may have invoked similar sub-parts of the lIFG in the early 350–550 ms time-window, yielding very similar ‘early’ P600-effects, and different, but potentially overlapping, sub-parts of the lIFG in the later 600–900 ms time-window, producing characteristically distinct P600-effects. In addition, the right hemisphere homologue of lIFG, the rIFG, has been shown to be active when processing complex stimuli [29], which may also affect the scalp distribution of the P600. A categorization of different instances of the P600 and the different circumstances under which they are elicited, may help us to further unravel what kinds of processing, including pragmatic processing, constitute the creation of meaning.

Finally, the results of the experiments combined are consistent with the interpretation that the early negativity found in Experiment 1 is actually an ELAN reflecting a category violation. In Experiment 1, the question in the violation condition requires the answer to talk about two AGENTs. This makes it very likely that participants expected to get a verb instead of an adverb (e.g., “exuberantly”). This category violation may have lead to an ELAN. In Experiment 2, there is no such category violation, and we did not find an ELAN there. Steinhauer & Drury [24] have recently argued that the functional significance of the ELAN component, which they associate strongly with Friederici's syntax-first model of language processing, is still rather unclear. Our present results, however, suggest that there is some sort of early effect of a mismatch between linguistic input and expectation. We do not believe, however, that the existence of category violation effects per se makes it necessary to adopt syntax-first models, as also other models assume that the language comprehension system engages in some form of prediction [14], [26], [27].

## Materials and Methods

### Ethics statement

The treatment of the participants conformed to APA and BPS ethical standards. Participants gave written consent for participation. The protocol was approved by the *Medical Ethical Committee of the University Medical Center Groningen* (METc UMCG).

### Participants

#### Experiment 1

Eighteen undergraduate students from the University of Groningen (6 male, age-range 18–29, average 20) participated, receiving payment or course credits for taking part in the experiment. All were right-handed native speakers of Dutch with normal, uncorrected vision.

#### Experiment 2

Twenty participants (8 male, age-range 18–34, average 22) that were either university students or recent university graduates, volunteered to take part in the experiment. All were right-handed native speakers of Dutch with normal or corrected-to-normal vision.

### Materials and Design

#### Experiment 1

Besides the two kinds of experimental question-answer pairs (20 per condition) where the answer sentence contained an NP-coordination, there were 40 filler dialogues (20 with a neutral question and 20 with a two-topic question) in which the answer consisted of an S-coordinated sentence, such as in the answer to (8):

8. Q: Wat gebeurde er?/Wat deden de ridder en de hertog?

‘What happened?’/‘What did the knight and the duke do?’

A: De ridder bevocht de prins en de hertog vluchtte.

‘The knight fought the prince and the duke fled’.

The use of S-coordinated sentences as fillers should minimize the chance of participants developing processing strategies for the NP-coordinated sentences in the critical dialogues. In addition, there were 100 filler items from an unrelated experiment on the processing of relative clauses.

#### Experiment 2

In experiment 2, we adapted the critical sentences of experiment 1 so that they ended after the first NP of the NP-coordination (see example dialogues in (9)):

9. Q: Wat gebeurde er?/Wat deden de burgemeester en de wethouder?

‘What happened?’/‘What did the mayor and the alderman do?’

A: De burgemeester prees het raadslid.

‘The mayor praised the councilor.’

The S-coordination fillers were the same as in experiment 1. There were also 116 fillers from an unrelated experiment. In both experiments, lists were created using a Latin Square, with equal numbers of items occurring in each condition on each list, and no list containing more than one version of a given item. The order in which experimental and filler items appeared was determined semi-randomly (i.e., allowing maximally three experimental items in consecutive order, but never two consecutive items in the same condition) and was the same for all lists. Each lists was presented to an equal number of participants and each participant saw only one list.

### Procedure

#### Experiment 1

Participants were instructed to read each sentence for comprehension, and to respond to an occasional content question (by means of pressing yes/no buttons on a button box; 35 for the entire experiment; example: “What did the detective and the greengrocer do?”–question: “Was there a detective?”). At the beginning of each trial, a fixation mark appeared for 1 second. After that, the dialogues were presented word-by-word in the center of the screen. Each word remained on screen for 243 ms, followed by a blank screen of 243 ms. Between question and answer there was a pause of 729 ms. The experiment took about 100 min, including preparation.

#### Experiment 2

Participants were instructed to read each sentence for comprehension, and to judge the semantic relatedness of an occasional probe word to the preceding sentence (by means of pressing yes/no buttons on a computer keyboard; example: “The lackey spied upon the baroness and the lady-in-waiting screamed”–probe: “nobility?”). At the beginning of each trial, a fixation mark appeared for 500 ms. This fixation mark was followed by a blank screen of the same duration. After that, the dialogues were presented word-by-word in the center of the screen. Each word remained on the screen for 240 ms, followed by a blank screen of 240 ms. Between question and answer there was a pause of 960 ms. The experiment took about 100 min, including preparation.

### EEG recording parameters

#### Experiment 1

EEG activity was recorded by means of 20 tin electrodes mounted in an elastic cap: FP1, FP2, FZA, F7, F3, FZ, F4, F8, T7, C3, CZ, C4, T8, P7, P3, PZ, P4, P8, O1, and O2. Bipolar horizontal EOG was recorded between electrodes at the outer canthi; bipolar vertical EOG was recorded for both eyes. Electrode impedances were kept below 5 *k*Ω. EOG and EEG signals (referred to the average of the two mastoids) were sampled at 1000 Hz, amplified (EEG: 0.2 mV/V; EOG 0.5 mV/V; time constant: 10 sec.), and digitally low-pass filtered with a cut-off frequency of 30 Hz.

#### Experiment 2

EEG activity was recorder by means of 62 tin electrodes mounted in an elastic cap: FP1, FP2, F3, F4, C3, C4, P3, P4, O1, O2, F7, F8, T7, T8, P7, P8, AFZ, CZ, FZ, PZ, AF3, AF4, FC3, FC4, CP3, CP4, PO3, PO4, PO7, PO8, AF7, AF8, FPZ, OZ, FC1, FC2, C1, C2, FCZ, FT9, FT10, F5, F6, FC5, FC6, C5, C6, CP5, CP6, P1, P2, P5, P6, P9, P10, PO9, PO10, CPZ, POZ, O9, O10, and IZ. Bipolar EOG was recorded between electrodes at the outer canthi; bipolar vertical EOG was recorded for the left eye only. EOG and EEG signals (referred to the average of the two mastoids) were sampled at 250 Hz, amplified (EEG: 0.2 mV/V; EOG 0.5 mV/V; time constant: 10 sec.), and digitally band-pass filtered with a low cut-off frequency of 0.01 Hz and a high cut-off frequency of 50 Hz.

## Conclusions

Violating the strong dependency between questions and answers by omitting information that language users have asked for, invoked pragmatic processes that modulate the amplitude of the P600 component. We interpret this increased P600 amplitude as a reflection of increased effort in constructing a coherent representation of what is being communicated. Possibly, this involves the computation of what the addressee of the question wants to communicate by leaving out part of the answer, and adding this information to the unfolding representation of the linguistic input in order to create coherence. Our results add to the as-of-yet small range of pragmatic phenomena that modulate the amplitude of the P600 component. This marks the importance of the P600 as an index of making sense, both in discourse and in conversation.

## Supporting Information

Materials S1 “Questions Left Unanswered”: Stimulus Materials.
(XLSX)
